# O-JMeSH: creating a bilingual English-Japanese controlled vocabulary of MeSH UIDs through machine translation and mutual information

**DOI:** 10.5808/gi.21014

**Published:** 2021-09-30

**Authors:** Felipe Soares, Yuka Tateisi, Terue Takatsuki, Atsuko Yamaguchi

**Affiliations:** 1 Computer Science Department, The University of Sheffield, Western Bank, Sheffield S10 2TN, UK; 2 National Bioscience Database Center, Japan Science and Technology Agency, Tokyo 102-8666, Japan; 3 Database Center for Life Science, Research Organization of Information and Systems, Kashiwa 277-0871, Japan; 4 Graduate School of Integrative Science and Engineering, Tokyo City University, Tokyo 158-8557, Japan

**Keywords:** controlled vocabulary, COVID-19, Japanese, multilingualism, natural language processing, translation

## Abstract

Previous approaches to create a controlled vocabulary for Japanese have resorted to existing bilingual dictionary and transformation rules to allow such mappings. However, given the possible new terms introduced due to coronavirus disease 2019 (COVID-19) and the emphasis on respiratory and infection-related terms, coverage might not be guaranteed. We propose creating a Japanese bilingual controlled vocabulary based on MeSH terms assigned to COVID-19 related publications in this work. For such, we resorted to manual curation of several bilingual dictionaries and a computational approach based on machine translation of sentences containing such terms and the ranking of possible translations for the individual terms by mutual information. Our results show that we achieved nearly 99% occurrence coverage in LitCovid, while our computational approach presented average accuracy of 63.33% for all terms, and 84.51% for drugs and chemicals.

## Introduction

References in MEDLINE are indexed according to MeSH terms. MeSH is a controlled vocabulary meta-thesaurus composed of more than 27,000 hierarchically structured descriptors. At higher levels of structure, one can find broad titles (e.g., Diseases - C), while narrow levels contain more specific titles (e.g., Diseases caused by Viruses - C02, Hepatitis A - C02.440.420).

In addition to its use in PubMed, the MeSH vocabulary has been used in a variety of ways in many areas of scientific research, including information retrieval, text mining, citation analysis, education, and bioinformatics research. When applied to information retrieval, MeSH terminology and its indexing results have been used to build visualization tools [1] and to distinguish between homonymous authors [2,3]. Biomedical text mining also makes extensive use of indexing on MeSH terms and has been used in various tasks, such as summarization, document clustering, and syntactic disambiguation [4].

Despite its large usage in natural language processing (NLP) tasks in English, MeSH is translated to a few other languages, such as Spanish, Portuguese, and French. However, some underrepresented languages on biomedical NLP have incomplete or outdated versions of MeSH, which is the case of Japanese. Thus, the ability to generate new open versions of MeSH in other languages, as well as improving the already existing ones, can help foster research in those languages.

In this application note, we propose the creation of a Japanese MeSH by combining different glossaries and exploring automatic translation. As a proof-of-concept, we used the LitCovid dataset [5], focused on coronavirus disease 2019 (COVID-19) research. We used automatic translation of sentences containing terms of interest and term selection via pointwise mutual information.

## Methods

Previous approaches to create dictionaries or extract parallel phrases for Japanese have resorted to existing bilingual dictionary and transformation rules to allow such mappings [6], or word alignment [7]. However, given the possible new terms introduced due to COVID-19 and the emphasis on respiratory and infection-related terms, we want to take advantage of the already mapped terms in English and the use of automatic translation.

A straightforward approach would be to directly translate the terms from English to Japanese using commercially available translators, such as Google Translate or Bing. However, given past experiments, this course of action can result in ill-translated terms due to polysemy and lack of context for single tokens [8]. One of the reasons for such behavior is that modern machine translation systems take huge advantage of context in a sentence to make the translation of a single token.

Thus, in our computational approach, we make use of full sentences in English that contain a specific desired MeSH term. After translation to Japanese, the equivalent in the target language will be found. This task can be described as the construction of bilingual dictionaries from parallel data [9].

However, since translating just one sentence for each term might lead to noisy results (that is, one may select a sentence that uses a non-standard or ambiguous translation for a given term), we will look for a set of sentences containing the same English MeSH term and then translate them to Japanese. By collecting multiple sentences, we expect that possible non-standard translations will be given less importance. We will extract the bilingual matching using pointwise positive mutual information (PPMI) [10]. Table 1 shows an example of how the proposed computational approach works considering the term “pulmonary embolism.”

The steps for the implementation of the computational method for constructing the bilingual dictionary are as follows:

1. Retrieve the most frequent MeSH terms from LitCovid.

2. For each of the terms from 1, retrieve k sentences in English that contain the given term.

3. Use an MT system to get the translation from English to Japanese of the complete sentence.

4. From the Japanese translation, segment the Japanese tokens using MeCab.

5. From 4, compute all possible {*1:n*}-grams (e.g. if n=3, all 1-grams, 2-grams, and 3-grams).

6. Calculate the MeSH-by-ngrams occurrence counts (i.e. the counts for every n-gram for the terms selected in 1).

7.Using PPMI, as in [10], find the most likely Japanese n-gram for a given MeSH.

## Results

We selected the MeSH terms in LitCovid appearing at least 50 times, resulting in a total of 1,039 terms. From the selected terms, we found that around 79% could already be found on existing vocabularies.

When using the proposed computational approach of machine translation and pointwise mutual information, we found that it had a precision of 63.33%. Meanwhile, the precision on translating using Google Translate on the isolated terms (without being in a sentence) was of 57.42%. Considering only a subset of MeSH terms representing drug names (validated by KEGG Drugs), the precision increases to 84.51%, which was similar to Google Translate performance of 84.03% on individual terms. We hypothesize that the higher precision is caused by the fact that drug names tend to have less variability (low number of synonyms), thus are easily distinguishable. The overlap of the computational approach with the manual curation of other bilingual sources was 68.43%.

On manual error checking, we found that the computational approach often failed to include the broad term into a specific term. For instance, for most of the cancers, the actual “neoplasm” equivalent in Japanese, “腫瘍”, was missing, leaving only the specific organ. The same issue happened for infections, where the actual word “infection” or “viral” was missing on the Japanese part. When considering the nature of MI, which gives less importance to tokens that appear frequently in different groups (terms), this failure is not completely unexpected. As a form of alleviating this, one could create a set of handcrafted rules to pre-filter the candidate terms before using MI to select the most probable one.

Our final bilingual glossary, O-JMeSH, covers approximately 99% of the occurrences in LitCovid, with a coverage of nearly 69% of all MeSH UIDs in the database. Thus, we can see that by combining both manual curation and computational efforts, we can lessen the effort required to map the most frequent terms occurring in COVID-19 related literature to the Japanese language.

## Figures and Tables

**Table 1.** Example of the computational approach for the term “pulmonary embolism”

English	MT Japanese
Pulmonary embolism has a high prevalence in COVID-19 patients	肺塞栓症はCOVID-19患者で高い有病率を示す
Pulmonary embolism is shown to increase the risk of death	肺塞栓症は死亡リスクを高めることが示されている
The patient presented bilateral pulmonary embolism	症例は両側性肺塞栓症を呈した。

In the English column, we show three sentences where context is given regarding the term. On the right column, we show the machine translated version for Japanese, with the term identified in bold. In this case, the term “肺塞栓症” is identified as the correct translation without directly inferring that 肺 is “pulmonary/lung”, 塞栓 as “embolus”, and 症 as “illness”. However, in this case, due to specialized dictionaries and low polysemy, this term could be directly inferred without requiring context. COVID-19, coronavirus disease 2019.
